# Balloon kyphoplasty as palliative care for painful pathological spinal fracture followed by lung cancer metastasis: A cohort study

**DOI:** 10.3389/fsurg.2022.1081823

**Published:** 2023-01-17

**Authors:** Jun-xin Zhang, Zhuo-run Song, Jun Zou, Jun Ge, Hui-lin Yang

**Affiliations:** Department of Orthopedic Surgery, The First Affiliated Hospital of Soochow University, Suzhou, China

**Keywords:** lung cancer, spinal metastases, kyphoplasty, end-stage life quality, spinal augmentation

## Abstract

**Background:**

Pathological spine fractures caused by metastases of lung cancer have brought great suffering to patients. Percutaneous kyphoplasty (PKP) has been considered a preferred alternative for painful spinal metastases. The clinical efficacy and safety of PKP for metastatic spinal lesions are urgently to be evaluated.

**Methods:**

A cohort study was conducted on 54 cases with pathologic spine fractures caused by metastasis of lung cancer. The correction of kyphosis was assessed by the Cobb angle. The life dependence and quality of the patients were evaluated by the Barthel Index of activities of daily living (ADL) and the quality-adjusted life year (QALY). Patients' survival was carefully recorded.

**Results:**

PKP significantly corrected the kyphosis compared with conservative treatment. The ratio of moderate dependence after fracture was clearly increased by PKP. QALY indicated a better life quality brought by PKP. However, PKP could not improve the survival rate of patients.

**Conclusion:**

PKP can be used as an effective palliative care treatment for patients with metastatic pathologic spinal fractures of lung cancer.

## Introduction

Cancer has been a major challenge to global health. Among the numerous types of cancer, lung cancer has gained widespread attention because of its high incidence and mortality rate. Bone is the most frequent site of distant metastases of lung cancer, especially the spine, femur, ribs, and sternum (1). Although 50% of bone metastases of lung cancer are asymptomatic, they often lead to serious complications (2). Pathological fractures, characterized by severe pain and loss of motility, have brought great suffering to patients with end-stage lung cancer.

Different from the obvious limitation of movement after pathological fractures of extremities, pathologic spine fractures are usually featured with prolonged back pain. Conservative analgesic treatment, radiotherapy, chemotherapy, and other means hardly achieve satisfactory clinical results for pathologic spine fracture patients (3). Besides, many patients are left with kyphosis despite the relieved pain. The compression on the abdominal organs and lungs caused by excessive kyphosis decreases appetite and affects pulmonary function (4). Due to cachexia status and lower life expectancy of end-stage patients, extensive open surgery may not be tolerated and would significantly increase mortality (5). With the development of minimally invasive surgery, percutaneous vertebral augmentation has been considered a preferred alternative for painful spinal metastases. Percutaneous vertebral augmentation includes percutaneous vertebroplasty (PVP) and percutaneous kyphoplasty (PKP). Compared with PVP, PKP is more effective in restoring kyphosis due to the propulsive effect of the balloon (6). With its rapid relief for patients, PKP appears to be required for patients with painful pathological spinal fracture.

However, there have been few reports on PKP in the treatment of lung cancer with spinal metastatic pathological fractures so far. The safety and the long-term efficacy of PKP in treating spinal metastases from lung cancer need to be further evaluated. More importantly, the improvement of end-stage patients' life quality by PKP needs to be further explored.

In this study, a cohort study was conducted on 54 cases with pathologic fractures caused by spinal metastasis of lung cancer to comprehensively evaluate the clinical efficacy and safety of PKP for metastatic spinal lesions.

## Materials and methods

### Study population

From January 2016 to December 2019, 54 patients with back pain were diagnosed as lung cancer metastatic spinal pathological fractures (Table 1). There were 28 patients (13 males and 11 females) with 37 vertebrae receiving PKP treatment, average age: 64.36 (range: 49–82). Twenty-six patients with 41 vertebrae were treated conservatively. The mean age was 60.78 (range: 31–83). Detailed fractured segments can be found in Table 2.

**Table 1:** Basic information of the patients enrolled.

Items		Non-KP	KP	Sig.
Age	60.31 ± 11.05	64.96 ± 9.40	0.644
Gender	Male	15	15	0.756
	Female	11	13	

**Table 2:** Fractured segments of the patients enrolled.

Segment	Non-KP (N = 26)	KP (N = 28)
T2	1	0
T3	2	0
T4	3	0
T5	3	0
T6	1	0
T7	3	2
T8	1	2
T9	3	1
T10	2	3
T11	3	5
T12	4	5
L1	4	6
L2	5	2
L3	2	5
L4	4	4
L5	0	2
Sum	41	37

### Participants

The diagnostic criteria of spinal metastases are as follows: Lung cancer patients with back pain were admitted to the hospital. The magnetic resonance imaging (MRI) examination showed a high signal of the vertebral body and pedicle on T2 phase and short time inversion recovery (STIR) phase, indicating a vertebral fracture. Furthermore, emission computed tomography (ECT) showed high metabolism activity of the spinal lesion.

**Figure 1:**
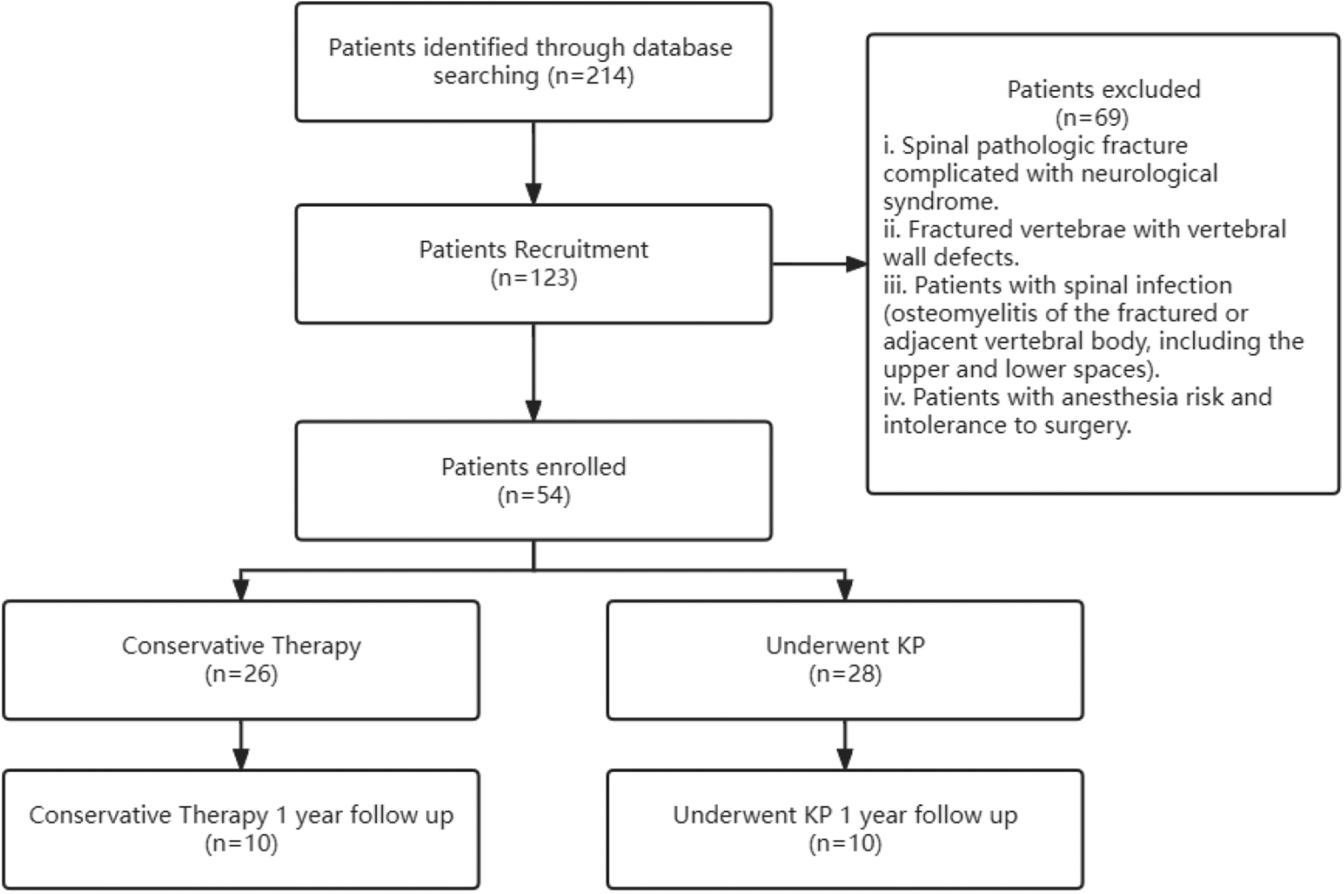
The flowchart of patient eligibility screening and follow-up.

All patients presenting with metastatic vertebral fractures were evaluated by a multidisciplinary team that included an experienced oncologist, radiologist, and orthopedic surgeon. PKP is only administered to patients with severe pain who have failed conservative treatment. X-ray and CT scan are performed to assess the integrity of the vertebral wall before the operation. Careful physical examination is required to identify the responsible vertebrae and to compare them with MRI results for confirmation. The flowchart of patient eligibility screening and follow-up has been shown in Figure 1.

### Exclusion criteria

(i) Pathologic fracture of the spine complicated by neurological syndrome. (ii) Fractured vertebrae with vertebral wall defects. (iii) Patients with spinal infection (osteomyelitis of the fractured or adjacent vertebral body, including the upper and lower spaces). (iv) Patients at risk of anesthesia and surgery.

### Surgical procedure and postoperative treatment

After obtaining informed consent, PKP was performed under general anesthesia. Kyphoplasty was performed according to the procedure described by Yang et al. Briefly speaking, the patient was placed in the prone position, with pads beneath the upper chest and pelvic regions. The insertion position was determined by preoperative fluoroscopic guidance. Pedicle channels were carefully established under C-arm fluoroscopic guidance. Specimens were taken from the working channel for biopsy prior to balloon inflation and cement injection. The balloon was then inflated to restore kyphosis. Poly (methyl methacrylate) (PMMA, Heraeus Medical GmbH, Germany) was injected, and the volume of cement used was 3.70 ml per vertebrae (range 1.5–6.0 ml). No surgery was stopped because of patient intolerance or cement leakage. Patients were encouraged to mobilize 6 h after the operation and were discharged or transferred to the rehabilitation department the next day.

All patients were treated with radiotherapy and chemotherapy for lung cancer. All the patients received bone preservation therapy.

### Radiographic assessment

X-ray examinations were obtained preoperatively, 3 days, 1 month, 3 months, and 1 year after treatment. Kyphosis was mainly determined by the Cobb angle on lateral radiographs. The Cobb angle is the angle between the upper endplate of the uninvolved vertebra above the fractured level and the lower endplate of the uninvolved vertebra below the fractured level. The anterior margin of vertebral height was measured. The collapse of the fractured vertebral body can lead to an increase in Cobb angle and a decrease in vertebral height.

### Barthel Index of ADL

The quality of life of patients was assessed by the Barthel Index of ADL (Table 3). The grading system is as follows: Grade I 100, no need for personal care. Grade II, 61–99, mildly dependent, requiring little help in daily life; Grade III, 41–60, moderately dependent, requiring care for most of daily life; Grade IV, ≤40, highly dependent, requiring care for daily life. Grades I and II are considered moderately dependent. Ration of Moderate Dependence = Months of Moderate Dependence/Life Months.

**Table 3:** Rating scale of Barthel Index of ADL.

Test Item	Criteria	Score
Bathing	Independent	5
	Dependent	0
Grooming	Independent	5
	Need help with personal care	0
Feeding	Independent	10
	Need help cutting, spreading, etc.	5
	Unable/gastric tube Indwelling	0
Dressing	Independent	10
	Need help but can do about half unaided	5
	Dependent	0
Toilet use	Independent	10
	Need some help, but can do something alone	5
	Dependent	0
Bowels	Continent	10
	Occasional accident	5
	Incontinent	0
Bladder	Continent	10
	Occasional accident	5
	Incontinent/catheterized	0
Stairs	Independent	10
	Need help	5
	Unable	0
Transfer (bed to chair and back)	Independent	15
	Minor help	10
	Major help, can sit	5
	Unable, no sitting balance	0
Mobility	Independent, >45 m	15
	Walk with help of one person, >45 m	10
	Wheelchair independent, including corners, >45 m	5
	Immobile or <45 m	0

### Quality-adjusted life year

QALY = Utility value of health status (%) × Life Years. The utility value of health status was directly measured by asking patients to rate their health status on a scale between 0 = dead and 100 = perfect health.

### Statistical analysis

All data were presented as mean ± standard deviation (SD). Independent samples *t* tests were used to compare the numerical parameters of patients with equal variance. The Chi-square test of independence is used for categorical variables. Rank variables were tested by the Kruskal–Wallis test. The results were considered significant when the *P* value was less than 0.05. All statistics were performed using SPSS 19.0 software.

## Results

No significance on age and gender was found between the enrolled KP group and the enrolled non-KP group (Table 1), neither does the pre-operation Cobb angles and vertebral height (Figures 3A,C). The life quality before fracture has no significance between the two groups (Figure 5B). About 20 patients out of 54 met the 1-year-follow-up and no significant differences were found in gender or age (Table 4). There was no loss of follow-up. There were four patients who survived in KP group till September 2022, and three in non-KP group. The QALY and Barthel Index of ADL results of the seven alive patients were excluded.

**Table 4:** Basic information of the patients who met the end point.

Items		Non-KP	KP	Sig.
Age	59.50 ± 11.617	66.60 ± 10.013	0.16
Gender	Male	5	4	0.653
	Female	5	6	

**Table 5:** Detailed information of radiographic assessment and life quality assessments.

		Non-KP	KP	Sig.
Radiographic assessment	Pre-Op Cobb angle (°)	7.43 ± 0.798	7.24 ± 0.677	0.321
	Post-Op Cobb angle (°)	8.33 ± 1.269	4.71 ± 0.787	0.020
	Pre-Op vertebral height (mm)	77.92 ± 12.332	76.07 ± 10.594	0.685
	Post-Op vertebral height (mm)	71.92 ± 14.337	83.64 ± 11.291	0.028
Life quality assessments	Barthel Index of ADL (Pre-Op)	65.38 ± 11.741	64.07 ± 8.533	0.638
	Barthel Index of ADL (3 months)	62.20 ± 23.875	78.61 ± 16.902	0.028
	Barthel Index of ADL (6 months)	61.83 ± 16.716	76.43 ± 17.492	0.041
	Barthel Index of ADL (12 months)	43.60 ± 30.888	75.00 ± 16.118	0.011
	QALY	0.248 ± 0.071	0.633 ± 0.172	0.047
	Months of moderate dependence	3.65 ± 1.133	6.42 ± 1.929	0.228
	Ratio of moderate dependence (%)	29.13 ± 5.655	67.29 ± 5.603	0.000

**Figure 2:**
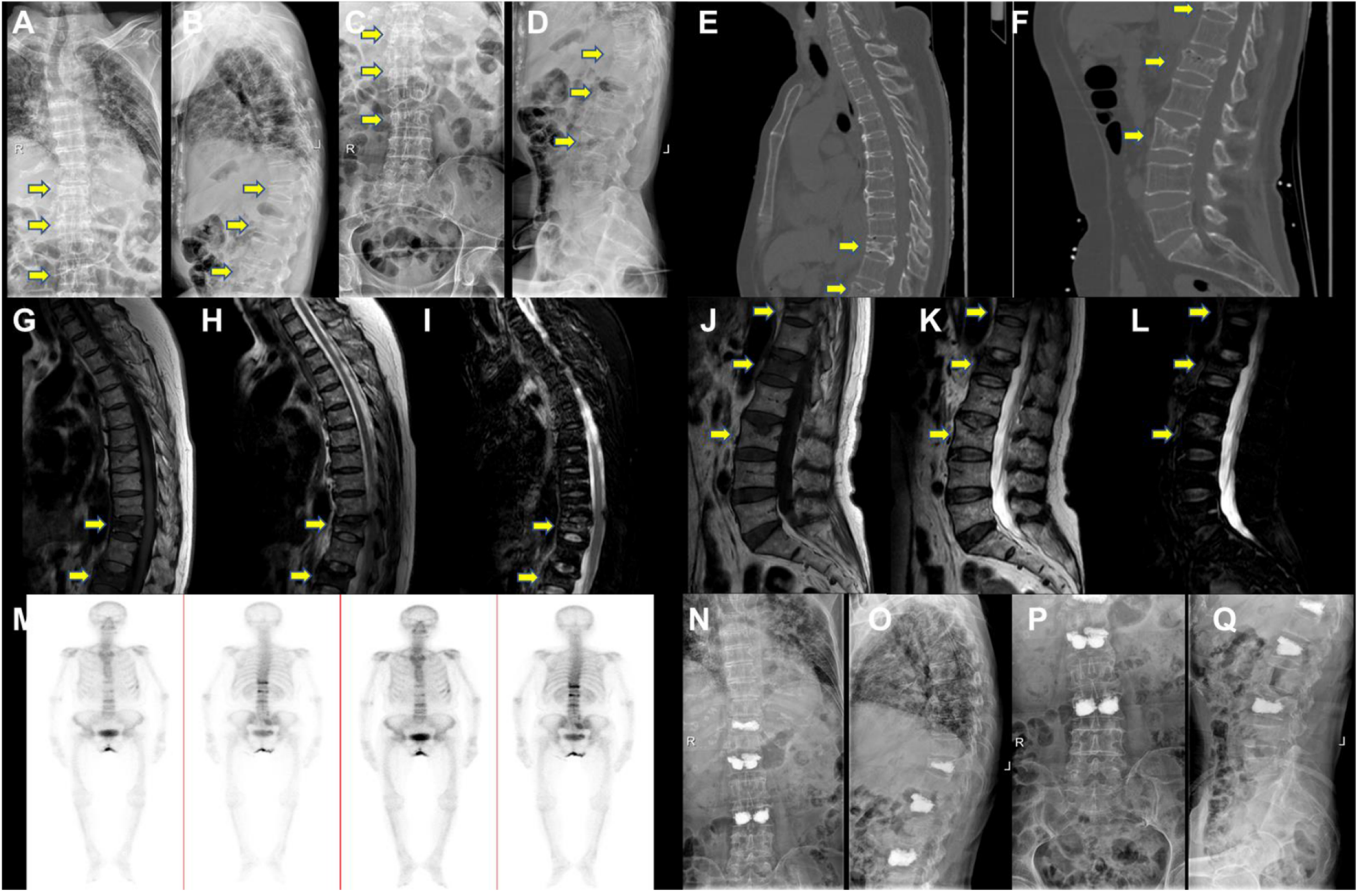
The representative patient's medical images. Pre-Op digital radiography (DR) images of (**A,B**) thoracic and (**C,D**) lumbar. Representative CT images of (**E**) thoracic and (**F**) lumbar. Representative MRI images of (**G**) T1, (**H**) T2, and (**I**) STIR. Thoracic and (**J**) T1, (**K**) T2, (**L**) STIR lumbar (**M**) and ECT image. Post-Op DR images of (**N,O**) thoracic and (**P,Q**) lumbar. Yellow arrows point to the injured vertebral bodies. ECT, emission computed tomography.

**Figure 3:**
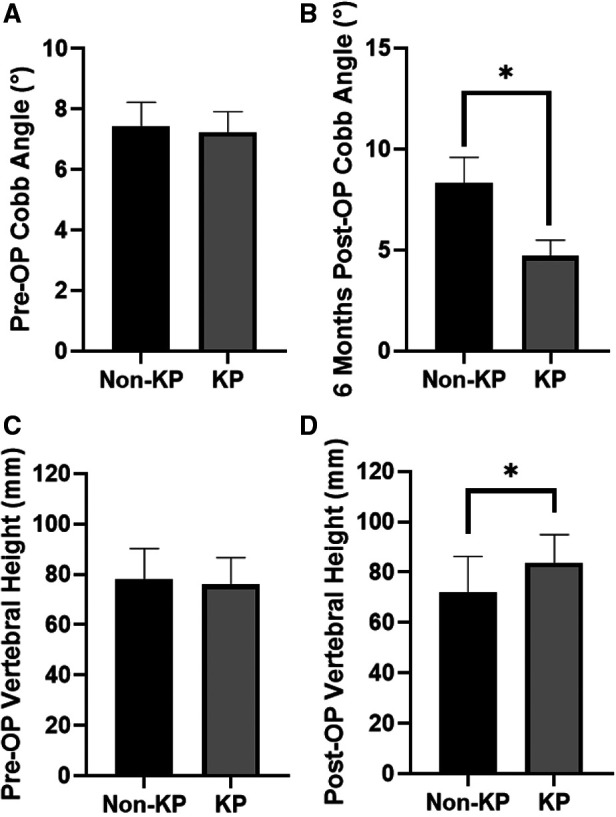
Cobb angle. (**A**) Before KP operation. (**B**) Six months after KP operation. Anterior height of vertebrates. (**C**) Before KP operation. (**D**) Six months after KP operation (*p < 0.05).

PKP procedures were successfully performed in all the patients in the KP group. The medical images of the representative patient were shown in Figure 2. The mean time of the surgical procedure for each segment was 38.10 ± 11.32 min (range: 19–55). No complications were found, including intraoperative cement leakage, bleeding, spinal cord injury, infection, pulmonary embolism, bone cement allergy, etc. The Cobb angle 6 months after the operation indicates a significant decrease in KP group (*p* < 0.05, Figure 3B and Table 5). Moreover, the vertebral heights of KP patients were significantly increased compared with conservative therapy (*p* < 0.05, Figure 3D and Table 5).

**Figure 4:**
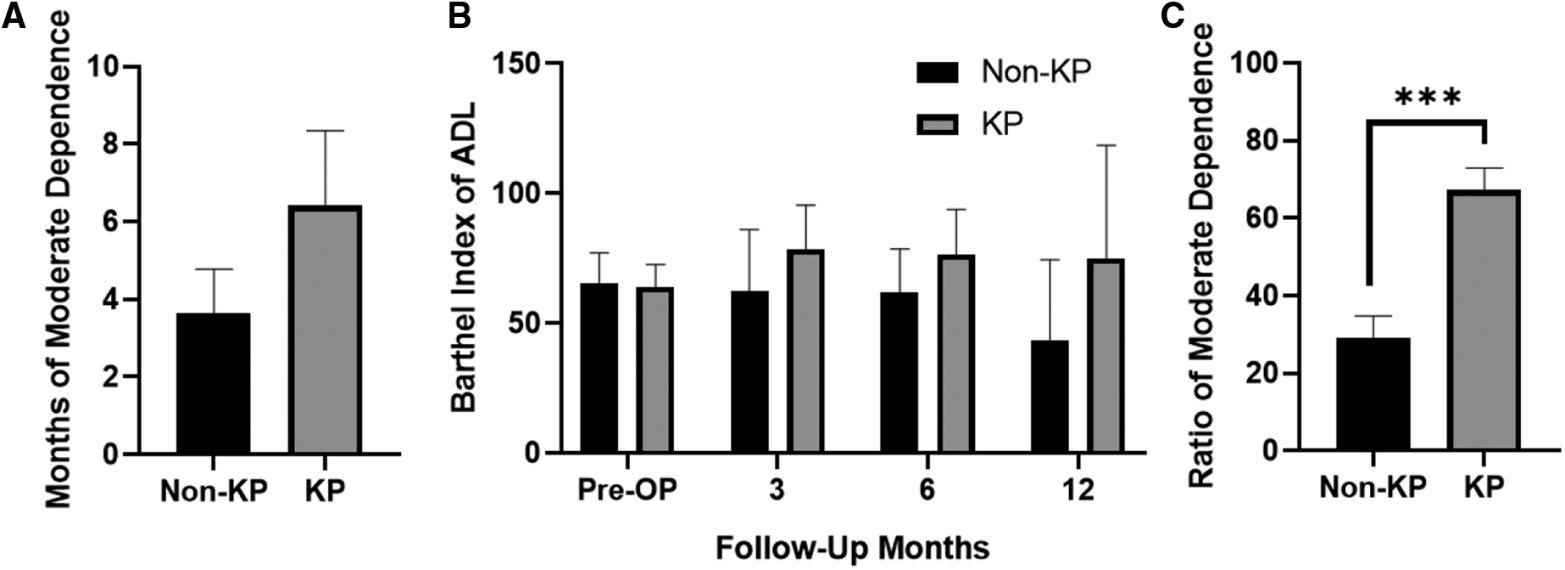
(**A**) Months of moderate dependence of the patients. (**B**) The absolute value of Barthel Index of ADL. (**C**) Ratio of moderate dependence in survival time of the patients (***p < 0.001).

**Figure 5:**
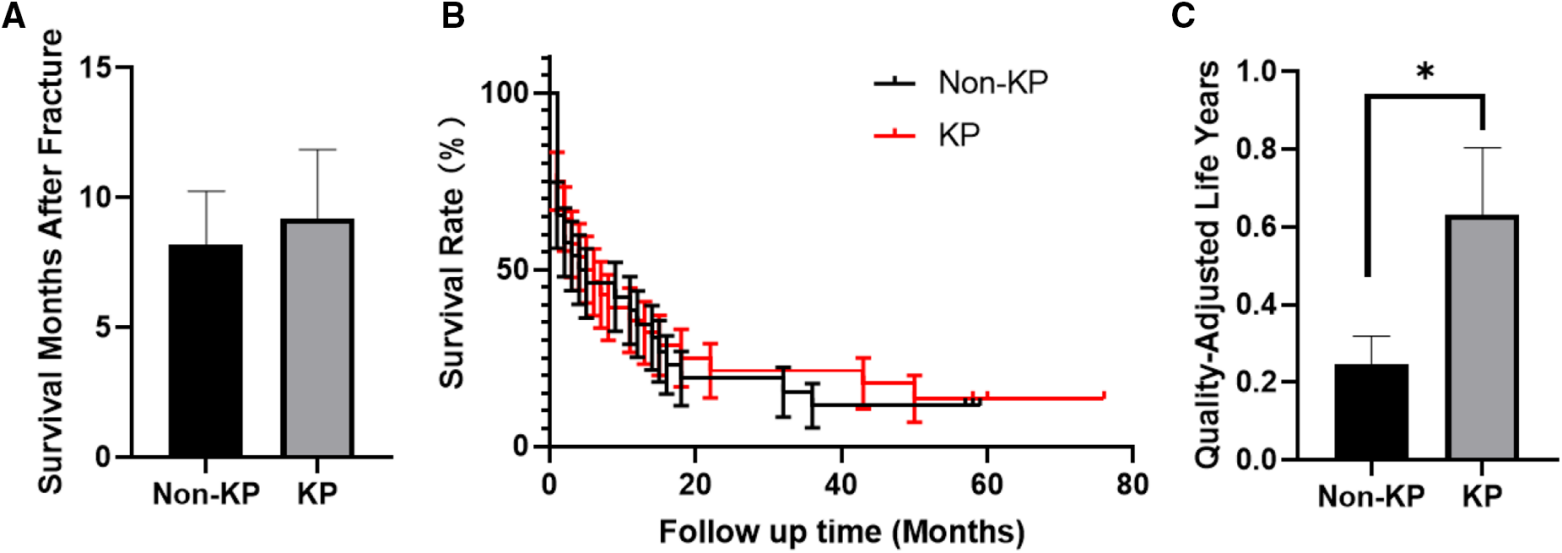
(**A**) Survival months of the patients after fracture. (**B**) Survival curve of the patients. (**C**) Quality-adjusted life years of the patients (*p < 0.05).

No significance on age and gender was found between the final-follow-up KP group and the final-follow-up non-KP group (Table 4). The month of moderate dependence of KP group was 6.42 ± 1.93 months, which is longer than that of non-KP group (3.65 ± 1.13), despite no statistical significance (*p* > 0.05, Figure 4A and Table 5). The KP group also showed a higher Barthel Index of ADL at the 3rd month, 6th month, and 12th month (Figure 4B and Table 5). Considering the survival time, KP has shown great advantages in improving the ratio of moderate dependence up to about 40% (*p* < 0.001, Figure 4C and Table 5), though KP cannot improve the survival rate and prolong the life year of the metastatic lung cancer patients (*p* > 0.05, Figures 5A,B). Surprisingly, KP clearly improved the QALYs of the patients compared with conservative treatment (*p* < 0.05, Figure 5C and Table 5).

## Discussion

Numerous studies have well demonstrated the clinical efficacy of vertebral augmentation (PVP and PKP) for vertebral compression fractures. The very first use of vertebral augmentation in tumors was reported by Galibert and Deramond in 1987 for the treatment of vertebral hemangiomas (7). Since then, several reports have revealed the great value of vertebral augmentation in treating painful spinal metastases (8, 9).

The purpose of PKP is to relieve pain and improve vertebral strength. Former studies have shown the excellent analgesic effect of PKP for spinal metastases (10). However, the analgesic mechanism of PKP remains unclear. On the one hand, PKP enhances the stability of vertebrae and reduces pain biomechanically (11). On the other hand, bone cements (PMMA) also play a role in killing cancer cells. The cytotoxicity of free monomers or polymeric exothermic reactions may destroy tumor cells and vertebral sensory nerves (12, 13).

Among all lung cancer patients with bone metastasis, most of them have osteolytic metastasis (14). Therefore, patients usually show a localized osteolytic lesion with trabecular bone destruction in the metastatic vertebrae. Considering osteolysis, pathological spinal fractures usually cause kyphosis and the subsequent complications. Compared with PVP, PKP is more effective in restoring kyphosis due to the propulsive effect of the balloon (6). Inspiringly, the mechanical support would not be affected by the tumor and the following therapies (radiotherapy and chemotherapy) (3, 10, 15, 16). Due to the major influence of kyphosis on pulmonary functions, a further increase in kyphosis may affect breathing and ultimately decrease the patient's end-stage life quality. Thus, restoring the local kyphosis angle is definitely helpful for patients with lung cancer. Consistent with the previous studies, our results revealed that PKP could effectively improve kyphosis, and the Cobb angles were significantly improved.

Notably, no complications were found in our surgical patients in the current study. The cavity created by the balloon makes it easy to inject relatively viscous bone cement, resulting in a low cement leakage incidence of PKP (17). Briefly speaking, PKP is a safe procedure for spinal metastases, but several tips should be emphasized. First, the integrity of the posterior vertebral wall should be carefully assessed by CT scan. Symptomatic spinal cord compression at the fractured vertebral segments is contraindicated for PKP. Cement leakage or tumor displacement into the spinal canal during injection would lead to severe complications. With the development of techniques, posterior wall or pedicle defects are no longer absolute contraindications for PKP (18). High-viscosity bone cements and appropriate balloon dilation are highly recommended. Second, if the tumor or fracture involves the pedicle, we propose to establish the pedicle channel through the healthy one. For lumbar vertebrae, a posterolateral extramedicular approach may be used instead of the transpedicular approach. While an extradicular approach between the rib head and the pedicle could be applied for thoracic vertebrae. Finally, the position of the balloon should be closer to the anterior wall compared with osteoporotic vertebral compression fracture, to avoid the displacement of tumor tissue to spinal cord or nerve.

Improving end-stage life quality is a crucial part of palliative care. Health-related quality of life refers to the measure of a patient's physical, psychological, and social functioning (19). The current study has demonstrated a longer time frame and a higher ratio of moderate dependence in the KP group. Despite no significance was found in absolute value, a higher ratio of moderate dependence of KP group has definitely indicated a better life quality of the metastatic lung cancer patients. The QALY is the product of life expectancy (estimated in years) and its quality over that time (estimated in utilities or quality of life units). It has been one of the most widely reported evaluations of health technology and its benefits (20). In the current study, PKP has increased the QALYs obviously compared with conservative therapy, further manifesting the satisfactory of the patients. Conservative management requires bed rest, resulting in acute bone mass loss and limited activity, not to mention bedsores and pulmonary infections caused by bed rest. Except for pain relief, the ultimate goal of PKP is to achieve a comprehensive treatment for cancer-related osteoporosis. Inspiringly, our results have proven that PKP can be used as effective palliative care for patients with end-stage diseases.

Unfortunately, PKP could not increase the survival time of patients, which may be due to the fact that PKP can only treat local cancer lesions and provide mechanical support but does not prevent the further metastasis and dissemination of primary lung cancer. However, we still believe that PKP is valuable considering the significant improvement in life quality of the end-stage patients.

There are some limitations in the current work. First, the sample size of patients enrolled was small. Second, the follow-up time was short. And more than half of the patients reached the ending-point during the follow-up interval. Finally, due to the limited number of cases, rigorous pairing failed to be achieved.

## Conclusion

Overall speaking, PKP can be an effective and safe solution for patients with metastatic lung cancer pathologic spinal fractures with persistent pain. Although it cannot improve the survival rate of patients, it can effectively improve their kyphosis, and, it can also improve the quality of their lives at the end stage. PKP should be considered a first-line treatment option for lung cancer metastatic pathologic spinal fractures.

## Data Availability

The raw data supporting the conclusions of this article will be made available by the authors, without undue reservation.
